# Caregiving Burden and Coping Strategies Among Informal Caregivers of Cancer Patients in Nigeria: From Duty to Distress

**DOI:** 10.3389/ijph.2025.1607735

**Published:** 2025-04-11

**Authors:** Nnabuike Celestine Eze, Chukwudi Gift Ezeugwu, Rachael Nwanezeobi Eze, Clara Ngozi Soronnadi, Chinonyelu Jennie Orji, Onyinye Hope Chime

**Affiliations:** ^1^ Department of Community Medicine, College of Medicine, Enugu State University of Science and Technology, Enugu, Nigeria; ^2^ Department of Human Physiology, College of Medicine, Enugu State Univerisity of Science and Technology, Enugu, Nigeria

**Keywords:** cancer caregiving burden, coping strategies, cancer patients, informal cancer caregivers, Enugu Nigeria

## Abstract

**Objectives:**

This study aims at determining the caregiving burden and the coping strategies adopted by informal caregivers of patients with cancer in tertiary health facilities in Enugu state.

**Methods:**

A cross-sectional study was conducted among 105 informal caregivers of cancer patients in tertiary health facilities in Enugu State using interviewer-administered questionnaires over 6 weeks. Data analysis was performed using SPSS version 27 and Chi square test of statistical significance was used to determine factors associated with caregiving burden and coping strategies.

**Results:**

The majority (27.6%) of caregivers were aged 41–50 years and females (62.9%). More than half (56.2) were not involved in the patients’ Activities of Daily Living (ADL). The most commonly utilized coping mechanisms was religion (92.4%) while behavioral disengagement (1%) and self-blame (1%) were the least utilized. Factors associated with caregiving burden include educational level, duration of patient’s illness and dependency of patient on caregiver.

**Conclusion:**

This study highlights the varying levels of caregiving burden and the predominant reliance on religious and acceptance-based coping strategies among informal caregivers in Enugu.

## Introduction

Cancer is a significant public health issue that affects people of all ages [1]. It is a leading cause of death globally, and serves as a significant barrier to increase in life expectancy in every country of the world [2]. Cancer is reported as the second most prevalent cause of death in developed countries and one of the top three causes of death in developing nations, accounting for an estimated 20% of all deaths globally [1, 3]. In 2020, there were reportedly about 19 million new cases and about 10 million fatalities worldwide [2]. In sub-Saharan Africa, 801,392 new cancer cases and 520,158 cancer-related deaths were reported whereas in Nigeria, 124,815 new cancer cases and 78,889 cancer-related deaths were estimated to have occurred [4, 5].

People living with cancer have several options for managing their disease, one of which is caregiving [6]. Caregiving refers to the regular care of someone, especially children, the elderly, the sick, or disabled individuals [7]. The person who offers this care is a caregiver [7, 8]. Cancer patients, due to their high morbidity and the chronic nature of their disease, receive multidimensional care from both formal and informal caregivers [9, 10]. This care which is provided throughout the different phases of cancer management includes monitoring treatment progress, managing symptoms related to treatment, drug administration, counseling, providing emotional, psychological, social, nutritional, financial, and spiritual support, and helping with personal and instrumental care [2, 9, 10].

Informal caregivers, despite the challenges they face, provide uncompensated care and are often underprepared and untrained to render such service [7, 9, 11–17]. They often have significant relationships with patients and include family members such as spouses, children, siblings, parents, friends, housekeepers, neighbors, members of the church, and partners [6–10, 18]. Most caregiving is provided at home by informal caregivers, often for financial constraints, instead of in a hospital or healthcare setting [9, 19–21].

Factors including westernization of diets and modifiable lifestyles, amongst other risk factors, has increased the prevalence of cancer in our setting [3, 22]. The likelihood of developing cancer is projected to increase by 85% in sub-Saharan Africa by 2027 [5, 22]. Yet, despite the rising burden of cancers in Africa, the availability of cancer screening and treatment services is limited [4]. Majority of cancer cases are diagnosed at advanced stages due to late presentation, amongst other factors. As a result, these patients require extensive care as the preferred treatment at such late stages is palliative and symptomatic. Additionally, the chronic progressive and incapacitating nature of this condition has a significant impact on both the patient and caregivers [23]. Unfortunately, these informal caregivers are often untrained, underprepared, or unprepared for such primary responsibilities [9, 11–17]. Unlike formal caregivers, who are professional healthcare providers paid or compensated for the services, informal caregivers are faced with a lot of burdens, including the financial and economic implications of cancer diagnosis and caregiving [9, 20–22].

Caregiver burden refers to caregivers’ distressing and burdensome challenges while caring for the sick [7, 24]. The caregiver burden is “the strain or load borne by a person who cares for a chronically ill, disabled, or elderly family member.” [24, 25] Caring for others can create discomfort due to the obligations and limitations it brings [24, 26]. These burdens could be physical, psychosocial, emotional, or financial [24]. Literature has revealed that informal caregivers are affected mainly by the burdens of cancer caregiving as most of the caregiving roles fall on them in the absence of formal caregivers [7, 27, 28]. This situation is exacerbated by delayed presentation, cultural beliefs surrounding illness and caregiving, inadequate treatment facilities, and unfavorable disease prognosis [28]. Given Nigeria’s severe economic circumstances and lack of functional health insurance, caregivers’ finances would likely be strained. Unfortunately, little attention is given to the informal caregivers’ health and welfare, which has worsened their burden.

Coping strategies and interventions refer to individuals’ behaviors and psychological means to deal with stress, challenges, or difficult emotions [7]. These coping mechanisms, including emotional-focused, problem-focused, and dysfunctional coping mechanisms, have been used by cancer caregivers to prevent caregiver burnout, a state of physical, emotional, and mental exhaustion, and to cope with the challenges they face [29, 30]. Coping strategies used by informal cancer caregivers are an area of concentration undeveloped in descriptive research and clinical intervention delivery [9, 28].

Studies on the burden of care associated with providing cancer care globally have mostly concentrated on the characteristics of the care burden experienced by professional/formal caregivers, rarely on relatives of cancer patients [9, 28]. Although caregiving has a proven negative impact on caregivers’ health, the majority of care delivery models place a strong emphasis on the needs and burdens of the patients. Literature reveals that no study on informal cancer burden has been conducted in Southeast Nigeria at the time of this study. However, in other settings, studies focused solely on the burden of caregiving, neglecting to explore the corresponding coping strategies. This study aims to determine the caregiving burden and the coping strategies adopted by informal caregivers of patients with cancer in tertiary health facilities in Enugu State.

## Methods

This study was conducted in tertiary health institutions in Enugu State, Southeast Nigeria, one of the six geopolitical zones in Nigeria. The state has four tertiary health institutions; three are publicly funded, and one is privately funded. The researchers selected two health institutions for this study: the University of Nigeria Teaching Hospital (UNTH) and the Enugu State University Teaching Hospital (ESUTH). UNTH Oncology Centre is multidisciplinary and effectively caters to the needs of cancer and other patients, as the Pain and Palliative Care Unit is affiliated with it, whose scope of services covers inpatients and outpatients of oncology and patients from different medical and surgical sub-specialties.

ESUTH does not have a stand-alone oncology unit. The Department of General Surgery manages cancer patients except for gynecological and childhood cancers, which the Obstetrics, Gynecology, and Pediatrics departments manage.

### Study Design and Population

This cross-sectional study aimed to identify the caregiving roles, burdens, and coping mechanisms of informal caregivers of cancer patients in tertiary health facilities in Enugu State. The study included all informal caregivers of cancer patients available throughout the study period who consented to participate in the study in both study centres. Both primary and secondary caregivers were included. Caregivers less than 18 years, caregivers of cancer patients without a confirmed cancer diagnosis based on histological studies and those with functional or cognitive impairments were excluded from the study.

### Data Collection and Analysis

Data was collected over 6 weeks using interviewer-administered questionnaires. Overall, 111 caregivers were eligible to participate in the study. Six declined participation in the study (2 from ESUTH and 4 from UNTH). Reasons for opting out include patient discouraging child (though an adult) from participating, stress, not interested, and secondary caregivers felt information provided by primary caregivers should suffice.

Validated tools, including the Katz Index of Independence in Activities of Daily Living (Katz-ADL), assess functional status in terms of the patient’s ability to perform self-care tasks independently. In this study, it was used to assess the roles played by the caregivers in patients’ ADL. The Katz-ADL comprised six essential activity items (bathing, dressing, toileting, transferring, continence, and feeding) [31]. Each patient’s dependency on the caregiver for their activities was scored on a scale of 0–6. A score of 0 indicates that the caregiver is not involved in the patient’s activities (patient is fully functional), 1 to 3 suggests moderate dependency (moderate impairment), and 4 to 6 indicates a high level of dependency (severe functional impairment).

Other tools include the Zarit Burden Interview (ZBI) scale, which assesses the caregivers’ psychosocial/emotional, financial/economic, and physical health burden. ZBI is a 22-item instrument measured on a 5-point scale: 0 = never, 1 = rarely, 2 = sometimes, 3 = frequently, and 4 = always [26]. This tool assesses the overall burden of informal caregiving. The total score ranges from 0 to 88, and the scores are categorized as follows: scores 0–20 indicate “no burden,” scores 21–40 “mild burden,” scores 41–60 “moderate burden,” and scores 61–88 “severe burden.”

The Brief COPE (Coping Orientations to Problems Experienced) tool explores the coping strategies caregivers of cancer patients utilized [32]. It consists of 28 items that measure 14 specific subscales, with two items for each subscale. These subscales include self-blame, behavioral disengagement, self-distraction, denial, substance use, emotional support, instrumental support, active coping, planning, acceptance, positive reframing, religion, venting, and humor. Respondents use a 4-point scale ranging from 1 (Never–I have not been doing this at all) to 4 (Frequently–I have been doing this a lot) to rate their use of each coping strategy in dealing with stressful events. Total scores for each scale are obtained by summing the relevant items for each scale. The scores for the scales are then categorized as follows: 2 to 4 = not utilized and 5 to 8 = utilized; the latter is further divided as 5 to 6 = poorly utilized and 7 to 8 = well-utilized.

The data was analyzed using Statistical Product and Service Solution (SPSS) version 27 [33] and presented in tables and charts as frequencies and proportions. Chi square test of statistical significance was used to determine factors associated with caregiving burden and coping strategies and the level of statistical significance was set at p-value of 0.05.

### Ethical Considerations

Ethical clearance and approval was obtained from the Health Research Ethics Committees of both institutions. Written informed consent was obtained from all individual participants in this study, having applied appropriate guidelines on issues such as human rights, safety and the confidentiality of the patients and caregivers.

## Results

Data were collected using interviewer-administered questionnaire from the two tertiary health facilities. Out of 111 potential respondents, only 105 participants were willing to participate in the study bringing the response rate to 94.6%. Majority of the respondents (78.1%) were recruited from UNTH. The results from the analysis are given in the tables below:

The age distribution shows that a majority (27.6%) of the caregivers were between 41–50 years with mean age of 41.63 ± 14.23 years. Most caregivers (62.9%) were females and had tertiary education (41.0%). Of these respondents, two-thirds (66.7%) were self-employed. Only 19 (18.1%) caregivers had underlying disease(s), the commonest being hypertension (36.84%) Table 1.

**TABLE 1:** Socio-Demographics characteristics of Cancer Caregivers in Enugu, Nigeria. (2024).

Variables	Frequency N = 105	Percentage (%)
Age (Years)		
18–30	26	24.8
31–40	24	22.9
41–50	29	27.6
>50	26	24.8
Mean ± SD	41.63 ± 14.23	
Gender		
Female	66	62.9
Male	39	37.1
Marital Status		
Married	67	63.8
Single	29	27.6
Widowed	9	8.6
Highest Level Of Education		
Primary (6 years)	19	18.1
Secondary (6 years)	39	37.1
Tertiary (4–6 years)	47	44.8
Employment Status		
Self-Employed	70	66.7
Salary-Earner	24	22.9
Unemployed	11	10.5
History of any Disease		
No	86	81.9
Yes	19	18.1
Type of disease^a^		
Hypertension	7	36.8
Diabetes	3	15.8
Peptic Ulcer Disease	3	15.8
Arthritis	2	10.5
Anxiety Disorder	1	5.3
Asthma	1	5.3
Chest Pain	1	5.3
Eye Pains	1	5.3
Leg Ulcer	1	5.3
Parkinsonism	1	5.3

^a^ Multiple response.

Table 2 shows that most caregivers were immediate (nuclear) family members of patients among which were children (26.7%) and siblings (20%). Most caregivers resided in same house with the patients (62.9%) and were aware of the patients’ conditions (76.2%). Among those aware of the patients’ conditions, 25% knew both the specific cancer, stage and treatment plans while 23.8% knew the condition as just cancer only. Majority of respondents (41.9%) had provided care for 1–6 months, with 39% of respondents spending at least 19 h daily providing care. Almost half (45.7%) of these caregivers always helped their patients, though majority of them (95.2%) were untrained to provide care and about two-third (60%) do not know the type of care their patients need Table 2.

**TABLE 2:** Relationship of caregivers to cancer patients in enugu, Nigeria. (2024).

Variable	Frequency N = 105	Percentage (%)
Relationship to Patient		
Child Parent Sibling Spouse Others	28 14 21 18 24	26.7 13.3 20.0 17.1 22.9
Reside In The Same House With Patient		
No	39	37.1
Yes	66	62.9
Level Of Information About Patient Condition		
Cancer Only (Or Chronic Disease)	19	23.8
Specific Cancer Only (site)	37	46.3
Specific Cancer + Stage (Metastatic Or Advanced)	4	5.0
Specific Cancer+/-Stage + Treatment	20	25.0
Duration of patient’s Illness		
≤6	44	41.9
7–12	19	18.1
13–18	11	10.5
≥19	31	29.5
Median (months)	8.0	
Duration Of Caregiving (Months)		
<1	17	16.2
1–6	44	41.9
7–12	12	11.4
13–18	11	10.5
≥19	21	20.0
Median (Months)	6.00	
Time/Duration Spent Caring For The Patient Daily (Hours)		
1–6	26	24.8
7–12	27	25.7
13–18	11	10.5
19–24	41	39.0
Mean (Hours)	14.92 ± 8.16	
Adequate Information about the Type of Care the Patient Needs		
No	63	60.0
Yes	42	40.0
Received/Receiving Any Training On How To Care For The Patient		
No	100	95.2
Yes	5	4.8
Frequency Of Help To Patient		
Always	48	45.7
Mostly	27	25.7
Some Of The Times	28	26.7
Rarely	2	1.9

Table 3 shows the caregiving roles of the caregivers in their patients’ activities of daily living (ADL). About 41% of the patients needed help bathing and dressing while a only 24.8% required to be fed by their caregivers. On categorization of ADL, more than half (56.2%) of the caregivers were not involved in patients’ ADL, while 38.1% were largely/fully involved Table 3.

**TABLE 3:** Caregiving roles–the Katz Index of independence in activities of daily living. Nigeria (2024).

Patient’s dependent daily activities on caregivers	Frequency (N = 105)	Percentage (%)
Bathing	43	41.0
Dressing	43	41.0
Toileting	40	38.1
Transferring	40	38.1
Continence	32	30.5
Feeding	26	24.8
Katz- ADL Dependency Index (Score Categorized)		
Caregiver not involved in patient’s ADL (0)	59	56.2
Caregiver moderately involved in patient’s ADL (1–3)	6	5.7
Caregiver largely involved in patient’s ADL (4–6)	40	38.1

Table 4 presents findings of the caregiver burden. Majority of the respondents (57.1%) sometimes feel they should be doing more for their relatives. Less than half of the respondents (49.5%) reported feeling stressed always between caring for their relatives and trying to meet other personal responsibilities and sometimes feel angry when around their relatives. Only 1 (1.0%) of the respondents always feel they will be unable to take care of their relatives much longer Table 4.

**TABLE 4:** Caregiving Burden among caregivers of cancer patients in Enugu, Nigeria. (2024) - Zarit Burden Interview.

S/No	Variables	Never (0)	Rarely (1)	Sometimes (2)	Frequently (3)	Always (4)
1.	Feel stressed between caring for your relatives and trying to meet other responsibilities for your family or work	6 (5.7)	12 (11.4)	18 (17.1)	17 (16.2)	52 (49.5)
2.	Feel embarrassed about your relative’s behavior	22 (21.0)	19 (18.1)	52 (49.5)	9 (8.6)	3 (2.9)
3.	Feel angry when you are around your relative	22 (21.0)	20 (19.0)	52 (49.5)	9 (8.6)	2 (1.9)
4.	Feel that your relative currently affects your relationship with other family members or friends in a negative way	30 (28.6)	37 (35.2)	28 (26.7)	8 (7.6)	2 (1.9)
5.	Afraid what the future holds for your relatives	20 (19.0)	6 (5.7)	30 (28.6)	29 (27.6)	20 (19.0)
6.	Feel strained when you are around your relatives	12 (11.4)	8 (7.6)	43 (41.0)	24 (22.9)	18 (17.1)
7.	Feel that you do not have as much privacy as you would like because of your relative	14 (13.3)	23 (21.9)	43 (41.0)	18 (17.1)	7 (6.7)
8.	Feel that your social life has suffered because you are caring for your relative	15 (14.3)	16 (15.2)	39 (37.1)	21 (20.0)	14 (13.3)
9.	Feel uncomfortable about having friends over because of your relative	31 (29.5)	30 (28.6)	36 (34.3)	5 (4.8)	3 (2.9)
10.	Feel that you have lost control of your life since your relative’s illness	15 (14.3)	18 (26.7)	32 (30.5)	23 (21.9)	7 (6.7)
11.	Wish you could just leave the care of your relative to someone else	28 (26.7)	31 (29.5)	27 (25.7)	14 (13.3)	5 (4.8)
12.	Feel uncertain about what to do about your relative	12 (11.4)	22 (21.0)	58 (55.2)	12 (11.4)	1 (1.0)
13.	Feel that you should be doing more for your relative	4 (3.8)	26 (24.8)	60 (57.1)	9 (8.6)	6 (5.7)
14.	Feel you could do a better job in caring for your relative	5 (4.8)	29 (27.6)	58 (55.2)	8 (7.6)	5 (4.8)
15.	Overall, how burdened do you feel in caring for your relative?	7 (6.7)	9 (8.6)	40 (38.1)	31 (29.5)	18 (17.1)
16.	Feel that your relative asks for more help than (s)he needs	17 (16.2)	34 (32.4)	35 (33.3)	13 (12.4)	6 (5.7)
17.	Feel that because of the time you spend with your relative that you do not have enough time for yourself	10 (9.5)	20 (19.0)	33 (31.4)	25 (23.8)	17 (16.2)
18.	Feel your relative is dependent upon you	21 (20.0)	33 (31.4)	17 (16.2)	17 (16.2)	17 (16.2)
19.	Feel your health has suffered because of your involvement with your relative	11 (10.5)	18 (17.1)	48 (45.7)	18 (17.1)	10 (9.5)
20.	Feel that your relative seems to expect you to take care of him/her as if you were the only one he/she could depend on	25 (23.8)	31 (29.5)	27 (25.7)	11 (10.5)	11 (10.5)
21.	Feel that you will be unable to take care of your relative much longer	45 (42.9)	26 (24.8)	31 (29.5)	2 (1.9)	1 (1.0)
22.	Feel that you do not have enough money to care for your relative in addition to the rest of your expenses	16 (15.2)	14 (13.3)	23 (21.9)	11 (12.5)	41 (39.0)

**FIGURE 1:**
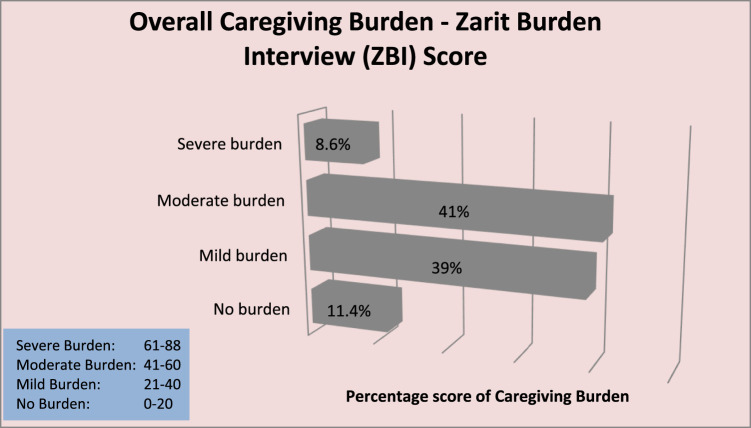
Bar chart displaying overall Caregiving Burden Score using the Zarit Burden Interview Tool (Enugu, Nigeria. 2024).

Figure 1 shows the overall burden of cancer caregiving of respondents. A high proportion of the respondents (88.6%) experienced one degree of burden or the other with 12 (11.4%) reporting no burden at all. Majority 43 (41%) had moderate burden, 41 (39%) had mild burden, and 9 (8.6%) severe burden.

The pattern of coping strategies utilized by caregivers in dealing with the burden of caregiving is shown in Table 5 (multiple responses). The commonest coping mechanisms utilized were religion (92.4%) and acceptance (85.7%), while the least utilized coping mechanisms were behavioral disengagement (1%) and self-blame (1%) Table 5.

**TABLE 5:** Coping Strategies/Mechanisms of Caregivers of Cancer patients in Enugu, Nigeria. (2024).

Coping Mechanisms	Not Utilized	Utilized		OverallUtilized
		Poorly Utilized	Well-Utilized	
Emotional-Focused				
Religion	8 (7.6)	30 (28.6)	67 (63.8)	97 (92.4)
Acceptance	15 (14.3)	46 (43.8)	44 (41.9)	90 (85.7)
Positive Reframing	36 (34.3)	58 (55.2)	11 (10.5)	69 (65.7)
Humor	76 (72.4)	19 (18.1)	10 (9.5)	29 (27.6)
Use of Emotional Support	22 (21.0)	59 (56.2)	24 (22.9)	83 (79.0)
Problem-Focused				
Instrumental Support	25 (23.8)	65 (61.9)	15 (14.3)	80 (76.2)
Active coping	31 (29.5)	54 (51.4)	20 (19.0)	74 (70.5)
Planning	42 (40.0)	55 (52.4)	8 (7.6)	63 (60.0)
Dysfunctional				
Self-Distraction	24 (22.9)	56 (53.3)	25 (23.8)	81 (77.1)
Denial	84 (80.0)	15 (14.3)	6 (5.7)	21 (20.0)
Venting	81 (77.1)	20 (19.0)	4 (3.8)	24 (22.9)
Self-Blame	104 (99.0)	0 (0.0)	1 (1.0)	1 (1.0)
Substance use	93 (88.6)	9 (8.6)	3 (2.9)	12 (11.4)
Behavioral disengagement	104 (99.0)	0 (0.0)	1 (1.0)	1 (1.0)

Not utilized: 2–4, Poorly utilized: 3–5, Well-Utilized: 7–8, Overall Utilized: 5–8.

Factors associated with caregiving burden include educational level (χ2 = 7.186, p = 0.028), duration of patient’s illness (χ2 = 4.186, p = 0.041) and dependency of patient on caregiver (χ2 = 5.344, p = 0.021) Supplementary File S1.

Supplementary Table S2 shows the factors associated with coping strategies among the respondents. A significantly higher proportion of respondents 41 years or less (74.5%) had better dysfunctional-focused coping strategies when compared with those above 41 years (χ2 = 6.681, p = 0.010). Respondents with high burden (68.7%) had higher emotional coping strategies than those with low burden (χ2 = 4.617, p = 0.032). Duration of illness (χ2 = 4.386, p = 0.036) and duration of caregiving (χ2 = 5.861, p = 0.015) were significantly associated with problem-focused coping strategy (Supplementary File S2).

## Discussion

The role and importance of informal caregivers in the management of their relatives or family members living with cancer are widely acknowledged. Findings from this study reveal that most caregivers are females (62.9%), in line with similar studies in Lagos (58%), Oyo (60%), Cross-River (63%) Nigeria; other African countries such as Uganda (60%), Namibia (86%) and the USA (73% and 58%) [2, 6, 23, 34–37]. Female predominance in caregiving is likely because, due to cultural roles, females are primarily assigned traditional caregiving roles, which promote their caregiving-related skills and abilities, making them more confident and self-assured when providing care. Also, the females spend more time at home than the males, who might be less available to provide care because they are more likely than the females to work full-time outside the house. Family members serve as frontline informal caregivers in this study. This is in line with other studies in Nigeria and India [38, 39].

Most caregivers in the present study were not trained to care for their relatives (95.2%). This finding is higher than 60% of caregivers who reported not to have received any training to aid them in caring for their cancer relatives in Lagos, Nigeria [2]. The disparity observed could be as a result of the fact that Lagos being a more cosmopolitan city has better access to information and more advanced healthcare facilities including caregiver resources unlike Enugu, though urbanized, may have a higher proportion of caregivers from semi-urban or rural areas. These findings suggest that many informal caregivers, who are usually unprepared for these roles, are trained while discharging their caregiving duties. In similar studies, most caregivers reportedly take on caring responsibilities without receiving enough training and are expected to handle caregiving obligations without much assistance, and this has resulted in detrimental/negative impact of their wellbeing [18, 23, 40]. This may increase the burden experienced while providing care and the quality of care they provide to their relatives.

Concerning caregivers’ involvement in patients’ ADL, the findings from this study reveal that a majority of the caregivers (56%) are not involved in the patient’s ADL, which means that most patients being cared for in this study were not functionally impaired. This finding is similar to a study in the USA where 60% of caregivers were involved in patients’ ADL [36]. However, contrary to expectations, higher caregiver involvement is expected in developing settings where there is limited availability of formal caregivers due to brain drain, with the expectation that informal caregivers take up most of the caregiving roles.

A high proportion of the respondents, 93 (88.6%), showed one degree of burden or the other, with only 8.6% severely burdened with the care. Similarly, participants from various studies in Nigeria felt burdened by their caregiving responsibilities, with rates ranging from 64.3% to 89.6% [2, 6, 23, 38]. This high burden reported in the present study could be attributed to the fact that most caregivers are closely related to the patients, reside in the same house, know about patients’ illnesses, and are unprepared/untrained to provide care. Also, most are self-employed, and their businesses suffer setbacks in their absence while caring for their loved ones. In some instances, the ones providing care are the breadwinners. Without a functional health insurance system in Nigeria, they are burdened with caring for their relatives and sourcing funds to pay medical bills. When asked about physical health burden, only 9.5% of the respondents were always burdened. This could invariably to linked to the fact that a higher proportion (56.2%) of the caregivers were not physically involved in patients’ ADL.

Despite the burden of caregiving, coping mechanisms encourage caregivers to persevere in their efforts and continue to provide care. Almost all caregivers (92%) utilized religious coping mechanisms. This is expected, given how deeply religious Africans, especially Nigerians are. Similar studies in Nigeria and the US support religion as caregivers’ most utilized coping mechanism [2, 30]. Fostering a positive relationship and placing “all reliance on God” assures caregivers that God is in charge of every situation [2]. The findings also imply that having faith in God assisted caregivers in accepting the situation as it is, seeing it as ‘the will of God.’ Thus, it is unsurprising that a high proportion (85.7%) accepted their fate. These mechanisms helps them cope with their stress effectively and is associated with greater life satisfaction [41]. Contrary to this view, using religion as a coping mechanism may result in suppression of frustration, discomfort or displeasure. The caregivers may find themselves isolating from exploring other non-religious coping strategies resulting in emotional exhaustion and instability overtime. Contrary findings were made in Namibia where majority of the caregivers utilized emotional support as a coping mechanism [37]. In this Namibian study, most caregivers experienced significant levels of distress. The pattern of coping mechanisms was significantly correlated with distress among the caregivers in this Namibian study, reflecting the high levels of psychosocial burden they experienced [37].

The least utilized coping mechanisms in this study were the dysfunctional mechanisms; self-blame and behavioural disengagement. This implies that the respondents in the present study were not overwhelmed by their caregiving experience. Instead, they actively engaged their burden, thereby gaining a sense of control over the situation. This could be attributed to low dependency of patients on their caregivers for their ADL, their religious beliefs, and the deep sense of responsibility for the patients, with the majority related to them. To avoid being judged as neglectful or not sympathetic, respondents may exaggerate feelings of satisfaction, resilience or coping. Furthermore, the Igbo culture, where most of the respondents belong to, expressing dissatisfaction or difficulty in caregiving may be perceived as a lack of compassion as communal support in the name of “*being their brother’s keeper*” is a duty. This support discourages any form of self-blame and disengagement. Acknowledging the cultural influence on caregiving can make caregivers feel respected and valued for their unique contributions.

Caregivers with low level of education was associated with caregiving burden. Low educational attainment may pose a challenge in comprehending medical terminologies, care protocols or accessing stress management services. They might be overwhelmed by the environment with a feeling of helplessness, resulting in withdrawal and emotional stress. This will invariably increase their burden. Also, caregivers whose patients had cancer for a longer duration had higher burden. Due to cultural and family dynamics, these caregivers could have been involved in patient’s care directly or indirectly resulting in the associated burden.

Although majority of the patients were not functionally impaired, caregivers whose patients were dependent on them had a higher burden. These patients depend on their caregivers for help with activities of daily living with the likelihood of such caregivers suffering exhaustion overtime. This dependency make limit the caregiver from attending to personal duties or engaging in other social activities which could relieve stress.

Caregivers with high burden in this study significantly had higher emotional coping strategies than those with low burden. They might rely on positive thinking, spirituality, or seek comfort from others. These caregivers could have developed this coping mechanism overtime to enable them bear the burden. It is not surprising that caregivers whose patients had been ill for longer duration and those who had cared for the patients for longer duration were significantly associated with problem-focused coping strategy. Adopting this practical approach to problem-solving is aimed at changing the demanding situation. Findings from this study reveal that being younger (less than 42 years) was associated with dysfunctional-focused coping strategies. This is similar to findings from a similar study in India where younger caregivers employed avoidant coping strategies more than older caregivers [39]. This they attributed to the fact that younger people are still developing their coping mechanisms unlike older ones with more established mechanisms [39].

### Strengths and Limitations

Our study is the first to investigate the caregiving burden and coping strategies of informal cancer caregivers in Southeast Nigeria, filling a gap in research on this topic in the region. Unlike other studies which mainly focused on the burden of caregiving, this study also explored the corresponding coping mechanisms used by informal caregivers. By highlighting the challenges faced by informal caregivers, this study has thrown more light on the burden of caregivers and has the potential to inspire changes in our understand and approach to these issues, ultimately leading to improved care and support for cancer patients. However, our study has some limitations. Being a cross-sectional study, this study only provides a snapshot in time and does not capture the dynamic, changing nature of caregiving, particularly as cancer progresses through different stages (initial, middle, long-term, and bereavement phases). Due to the sensitive nature of the study appears, caregivers may avoid honest reporting of feelings or coping to align with their perceived societal expectations. This social desirability bias was minimized as respondents were repeatedly assured their responses are confidential and anonymous. Determining the validity and reliability would have guaranteed cultural validation and tool adaptation for complete replicability in this context, even though the study instruments are standardized, validated, and utilized for comparable studies in Nigeria. The ZBI–22 tool used in this study did not fully explore the presence of psychosocial issues such as depression, anxiety, or mood disorders among the caregivers. Also, while financial burdens were acknowledged, the study did not provide an in-depth analysis of specific financial impacts, such as job loss or balancing caregiving responsibilities with personal and professional commitments.

These strengths and limitations help frame the study’s contribution to understanding caregiving burdens in the context of cancer in Nigeria, while also identifying areas for improvement in future research.

### Implications for Practice and Future Research

The findings of this study have practical implications for the care and support of cancer patients in Enugu State. The study provides valuable insights that could lead to changes in public health policy to improve the care and support for both cancer patients and their informal caregivers, particularly by addressing their burdens and providing better support mechanisms. Healthcare professionals in oncology departments should provide informal caregivers with sufficient information on the responsibilities of caregiving, the specific care needed by patients, and how to provide this care. Since many caregivers in this study are religious and use religious coping mechanisms, it may be beneficial for health professionals to collaborate with religious leaders to integrate religious practices into the caregiving support program; while religious leaders provide spiritual and moral support, healthcare providers will offer practical caregiving skills and psychological care. This holistic approach will provide opportunities for the caregiver to access professional training, counselling and spiritual support to cope with caregiving demands, thereby enhancing the effectiveness of a coping program. However, to avoid overstepping of roles, there is need for roles to be defined from the onset as religious leaders may inadvertently engage in providing medical advice.

Longitudinal cohort study, which would follow patients and their caregivers over an extended period, should be employed to provide a more comprehensive understanding of the impact of caregiving and adequately explore whether these coping mechanisms are effective in reducing burden over time or not. Future research should explore whether these strategies merely help caregivers endure stress or actively reduce the emotional, physical, and financial burdens they face.

### Conclusion

This manuscript addresses an important topic that has not been well-explored in Southeast Nigeria. Based on the findings, the majority of caregivers lived in the same household as the patients and were aware of their illnesses. They usually provided care for an average of 15 h per day over a period of 11 months. Most of these caregivers undertook this responsibility without being adequately prepared or trained for the type of care the patient needed. Nearly all of the caregivers reported feeling burdened, with about half of them describing their burden as moderate to severe. Majority of the caregivers utilized religion, acceptance, emotional and instrumental support, and self-distraction as coping mechanisms to reduce the stress and burden of caregiving. They were less likely to resort to dysfunctional coping mechanisms. The findings of this study can inspire potential changes in the way we understand and address the challenges faced by informal caregivers. This potential for policy changes is essential for improving the wellbeing of both caregivers and patients, enhancing the quality of care, and promoting equity in healthcare delivery.

## Data Availability

All information and resources, software, and custom code support claims and adhere to field standards.
