# Supplementary material for: Caregiving Burden and Coping Strategies Among Informal Caregivers of Cancer Patients in Nigeria: From Duty to Distress
Source: Int J Public Health. 2025 Apr 11;70:1607735. doi: 10.3389/ijph.2025.1607735 (PMC12021601; doi:10.3389/ijph.2025.1607735)
Supplement: Supplementary file 1 [file DataSheet1.pdf]

Table S 1: Factors associated with caregiving burden among caregivers of cancer patients in Enugu, Nigeria. (2024)

Variable	Low burden n=38	High burden n=67	X^2	p-value
**Age**				
≤41 years	19 (37.3)	32 (62.7)	0.049	0.825
> 41 years	19 (35.2)	35 (64.8)		
**Gender**				
Male	15 (38.5)	24 (61.5)	0.139	0.710
Female	23 (34.8)	43 (65.2)		
**Marital Status**				
Single	29 (38.2)	47 (61.8)	0.461	0.497
Married	9 (31.0)	20 (69.0)		
**Highest Educational Qualification**				
Primary	2 (10.5)	17 (89.5)	7.186	0.028*
Secondary	18 (46.2)	21 (53.8)		
Post-secondary	18 (38.3)	29 (61.7)		
**Employment status**				
Unemployed	6 (54.5)	5 (45.5)	5.346	0.069
Self-employed	20 (28.6)	50 (71.4)		
Salary earner	12 (50.0)	12 (50.0)		
**Resident in the same house**				
Yes	17 (43.6)	22 (56.4)	1.471	0.225
No	21 (31.8)	45 (68.2)		
**Duration of Patient's Illness**				
≤ 8 months	26 (44.8)	32 (55.2)	4.186	0.041*
> 8 months	12 (25.5)	35 (74.5)		
**Duration of Caregiving**				
≤ 6 months	25 (41.0)	36 (59.0)	1.448	0.229
> 6 months	13 (29.5)	31 (70.5)		
**ADL Dependency of patient on caregiver**				
Not Dependent	27 (45.8)	32 (54.2)	5.344	0.021*
Dependent	11 (23.9)	32 (76.1)		

* Statistically significant values

Table S 2: Factors associated with Coping strategies/mechanisms among caregivers of cancer patients in Enugu, Nigeria. (2024)

Variable	Emotional-Focused				Problem-Focused				Dysfunctional-Focused			
	Low coping n=41	High coping n=64	χ^2	p-value	Low coping n=43	High coping n=62	χ^2	p-value	Low coping n=40	High coping n=65	χ^2	p-value
**Age**												
≤41 years	18 (35.3)	33 (64.7)	0.587	0.444	19 (37.3)	32 (62.7)	0.561	0.454	13 (25.5)	38 (74.5)	6.681	0.010*
> 41 years	23 (42.6)	31 (57.4)			24 (44.4)	30 (55.6)			27 (50.0)	27 (50.0)		
**Gender**												
Male	17 (43.6)	22 (56.4)	0.538	0.463	17 (43.6)	22 (56.4)	0.178	0.673	11 (28.2)	28 (71.8)	2.573	0.109
Female	24 (36.4)	42 (63.6)			26 (39.4)	40 (60.6)			29 (43.9)	37 (56.1)		
**Marital Status**												
Single	30 (39.5)	46 (60.5)	0.021	0.885	30 (39.5)	46 (60.5)	0.249	0.618	33 (43.4)	43 (56.6)	3.310	0.069
Married	11 (37.9)	18 (62.1)			13 (44.8)	16 (55.2)			7 (24.1)	22 (75.9)		
**Highest Educational Qualification**												
Primary	8 (42.1)	11 (57.9)	1.824	0.402	10 (52.6)	9 (47.4)	4.467	0.107	10 (52.6)	9 (47.4)	4.372	0.112
Secondary	12 (30.8)	27 969.2)			19 (48.7)	20 (51.3)			17 (43.6)	22 (56.4)		
Post-secondary	21 (44.7)	26 (55.3)			14 (29.8)	33 (70.2)			13 (27.7)	34 972.3)		
**Employment status**												
Unemployed	5 (45.5)	6 (54.5)	0.981	0.612	6 (54.4)	5 (45.5)	1.010	0.604	5 (45.5)	6 (54.5)	2.328	0.312
Self-employed	25 (35.7)	45 (64.3)			27 (38.6)	43 (61.4)			29 (41.1)	41 (58.6)		
Salary earner	11 (45.8)	13 (54.2)			10 (41.7)	14 (58.3)			6 (25.0)	18 (75.0)		
**Resident in the same house**												

Yes	30 (45.5)	36 (54.5)	3.065	0.080	26 (39.4)	40 (60.6)	0.178	0.673	25 (37.9)	41 (62.1)	0.004	0.953
No	11 (28.2)	28 (71.8)			17 943.6)	22 (56.4)			15 (38.5)	24 (61.5)		
**Duration of Patient's Illness**												
≤ 8 months	25 (43.1)	33 (56.9)	0.896	0.344	29 (50.0)	29 (50.0)	4.386	0.036*	24 (41.1)	34 (58.6)	0.593	0.441
> 8 months	16 934.0)	31 (66.0)			14 (29.8)	33 (70.2)			16 (34.0)	31 (66.0)		
**Duration of Caregiving**												
≤ 6 months	26 (42.6)	35 (57.4)	0.782	0.377	31 (50.8)	30 (49.2)	5.861	0.015*	25 (41.0)	36 (59.0)	0.515	0.473
> 6 months	15 (34.1)	29 (65.9)			12 (27.3)	32 (72.7)			15 (34.1)	29 (65.9)		
**Caregiver received Training**												
Yes	1 (20.0)	4 (80.0)	0.646*	0.349	3 (60.0)	2 (40.0)	0.398*	0.331	2 (40.0)	3 (60.0)	1.000*	0.634
No	40 (40.0)	60 (60.0)			40 (40.0)	60 (60.0)			38 (38.0)	62 (62.0)		
**ADL Dependency of patient on caregiver**												
Not Dependent	26 (44.1)	33 (55.9)	1.426	0.232	23 (39.0)	36 (61.0)	0.216	0.642	24 (40.7)	35 (59.3)	0.381	0.537
Dependent	15 (32.6)	31 (67.4)			20 (43.5)	26 (56.5)			16 (34.8)	30 (65.2)		
**ZBL Burden Categorized**												
Low burden	20 (52.6)	18 (47.4)	4.617	0.032*	15 (39.5)	23 960.5)	0.054	0.816	17 (44.7)	21 (55.3)	1.114	0.291
High burden	21 (31.3)	46 (68.7)			28 (41.8)	39 (58.2)			23 (34.3)	44 (65.7)		

*FT
